# A systematic review and meta-analysis of phase III randomized controlled trials to assess the risk of pneumonia, URTIs, and VTE in multiple myeloma patients treated with isatuximab

**DOI:** 10.37349/etat.2025.1002300

**Published:** 2025-03-19

**Authors:** Daniel Thomas Jones, Hazem Aboaid, Ramaditya Srinivasmurthy, Kevin Nguyen, Rishi Kumar Nanda, Jason Ta, Benjamin Tzer-Ming Chuang, Yin Mon Myat, Aishwarya Hanspal, Kyaw Zin Thein, Thura Win Htut

**Affiliations:** University of Bologna, Italy; ^1^Touro University Nevada College of Osteopathic Medicine, Las Vegas, NV 89014, USA; ^2^Department of Internal Medicine, Kirk Kerkorian School of Medicine at UNLV, Las Vegas, NV 89106, USA; ^3^Department of Internal Medicine, St. Rose Dominican Hospital, San Martin Campus, Las Vegas, NV 89113, USA; ^4^Department of Internal Medicine, One Brooklyn Health—Interfaith Medical Center Campus, Brooklyn, NY 11213, USA; ^5^Department of Internal Medicine, Sunrise Health GME Consortium, Las Vegas, NV 89128, USA; ^6^Division of Hematology and Medical Oncology, Comprehensive Cancer Centers of Nevada, Central Valley, Las Vegas, NV 89169, USA; ^7^Department of Haematology, Aberdeen Royal Infirmary, AB25 2ZN Aberdeen, UK

**Keywords:** Multiple myeloma, isatuximab, pneumonia, upper respiratory tract infections (URTIs), venous thromboembolism (VTE), randomized controlled trials (RCTs), adverse events, hematologic malignancy

## Abstract

**Background:**

Multiple myeloma (MM) is a hematologic malignancy characterized by the clonal proliferation of malignant plasma cells in the bone marrow, constituting approximately 13% of all hematologic malignancies. Isatuximab is a monoclonal antibody targeting the CD38 protein on myeloma cells, causing cell death through various immune-mediated mechanisms. Clinical trials have shown that adding isatuximab to standard regimens for MM significantly enhances efficacy but introduces some notable toxicities. The purpose of this study is to determine the risk of pneumonia, upper respiratory tract infections (URTIs), and venous thromboembolism (VTE) in patients with MM treated with isatuximab.

**Methods:**

We conducted a comprehensive literature search using Medline, Embase, and Cochrane databases from inception through July 22nd, 2024. Phase III randomized controlled trials (RCTs) utilizing isatuximab in newly diagnosed MM (NDMM) and relapsed and refractory MM (RRMM) reporting pneumonia, URTIs, and VTE as adverse events were included. Mantel-Haenszel (MH) method was used to calculate the estimated pooled risk ratio (RR) with 95% confidence interval (CI). Heterogeneity was assessed with Cochran’s Q-statistic. Random effects model was applied.

**Results:**

A total of 1,044 patients from three phase III RCTs (ICARIA-MM, IKEMA, IMROZ) were included for pneumonia and URTI analysis, while 1,403 patients from three trials (IKEMA, IMROZ, GMMG-HD7) were included for VTE evaluation. The incidence of any-grade pneumonia was higher in the isatuximab group (30.1% vs. 23.2%; RR, 1.31; 95% CI 1.06–1.61; *P* = 0.01), as was high-grade pneumonia (20.8% vs. 15.3%; RR, 1.38; 95% CI 1.06–1.81; *P* = 0.02). No statistically significant differences were observed between the isatuximab and control groups for any-grade URTIs, high-grade URTIs, or VTE.

**Discussion:**

This meta-analysis highlights a significant increase in the incidence of pneumonia with the addition of isatuximab to standard myeloma regimens, underscoring the need for routine antibiotic prophylaxis, thromboprophylaxis, vigilant monitoring and early intervention to mitigate these risks.

## Introduction

Multiple myeloma (MM) is a clonal plasma cell malignancy accounting for approximately 13% of hematologic malignancies [1]. It is marked by significant bone marrow infiltration, immune dysregulation, and end-organ damage, including renal failure, hypercalcemia, and osteolysis, which contribute to substantial morbidity and mortality [2]. Despite advancements in therapeutic strategies such as proteasome inhibitors, immunomodulatory drugs, and monoclonal antibodies, MM remains a largely incurable disease. The high relapse rates associated with MM necessitate continual innovation to improve patient outcomes and address unmet clinical needs [2, 3].

CD38-targeting monoclonal antibodies, including isatuximab and daratumumab, have significantly advanced MM therapy [4]. Both antibodies target the CD38 glycoprotein expressed on plasma cells and leverage immune-mediated mechanisms such as antibody-dependent cell-mediated cytotoxicity and complement-dependent cytotoxicity. However, isatuximab differs from daratumumab in its epitope binding and ability to induce direct apoptosis independent of complement-dependent cytotoxicity, which may enhance its efficacy [5]. Clinically, isatuximab has demonstrated comparable or superior outcomes when combined with standard regimens such as pomalidomide or carfilzomib. However, the distinct pharmacokinetics and immune engagement profiles of isatuximab warrant further investigation, particularly regarding its safety profile [6].

Infections, including pneumonia and upper upper respiratory tract infections (URTIs), as well as venous thromboembolism (VTE) events, are common and significant complications in MM [7]. Pneumonia, in particular, remains a leading cause of morbidity and mortality, often necessitating hospitalization and intensive care. This risk is amplified by the profound immune dysfunction inherent to MM and further worsened by its treatments, underscoring the importance of vigilant monitoring and preventive strategies [8]. Similarly, VTE is a major contributor to morbidity and mortality, with an incidence exceeding 10% over the course of the disease. The introduction of therapies such as immunomodulatory drugs and proteasome inhibitors has further changed the epidemiology of thrombotic events, revealing an urgent need for optimized thromboprophylaxis [9]. These risks are compounded by the disease itself, the immunosuppressive effects of treatment, and the introduction of novel agents like isatuximab [6]. While prior meta-analyses, including our 2023 study, have characterized the safety profile of isatuximab in patients with relapsed and refractory MM (RRMM), they have been limited to RRMM populations and earlier clinical trials [10]. This study builds on that work by incorporating data from a newly available 2024 trial and expanding the analysis to include both newly diagnosed MM (NDMM) and RRMM patients. By systematically evaluating the incidence of pneumonia, VTE, and other adverse events across these broader populations, this study aims to provide a more comprehensive understanding of the safety profile of isatuximab, thereby addressing an important gap in the literature.

This study aims to quantify the incidence of pneumonia, URTIs, and VTE in MM patients treated with isatuximab. By providing a comprehensive evaluation of its safety profile, this study seeks to inform clinical practice and support the safe integration of isatuximab into MM treatment regimens.

## Materials and methods

The systematic review and meta-analysis were conducted in accordance with the Cochrane Handbook for Systematic Reviews of Interventions and adhered to the Preferred Reporting Items for Systematic Reviews and Meta-Analyses (PRISMA) guidelines. The primary objective was to evaluate the incidence of pneumonia, URTIs, and VTE in MM patients treated with isatuximab in combination with standard therapies compared to control groups receiving standard therapies alone. A comprehensive literature search was performed using the keywords “isatuximab” AND “multiple myeloma” across Medline, Embase, and Cochrane databases from inception through July 22, 2024. Additional searches included conference proceedings and meeting abstracts from major hematology and oncology societies to identify unpublished data. The search was limited to human studies and randomized controlled trials (RCTs). Non-English studies were excluded.

The inclusion criteria for this analysis were phase III RCTs evaluating isatuximab in combination with standard therapies for NDMM or RRMM and reporting pneumonia, URTIs, or VTE as adverse events. Phase III trials were exclusively selected to deliver the highest standard of evidence on intervention efficacy and safety, utilizing larger, more diverse patient cohorts within stringent methodological frameworks. This approach minimized dataset heterogeneity, optimizing the statistical power and reliability of the meta-analysis.

Both the treatment and control groups in the included trials received standard prophylactic antibiotics and thromboprophylaxis with either aspirin or low molecular weight heparin (LMWH) to mitigate baseline infection and thrombosis risks. The control groups were carefully matched with the treatment groups in terms of baseline risk factors, including age, disease stage, comorbidities, and prior treatments, to ensure comparability and minimize confounding variables. This approach enhances the reliability of the comparative safety analysis between the study arms.

**Figure 1:**
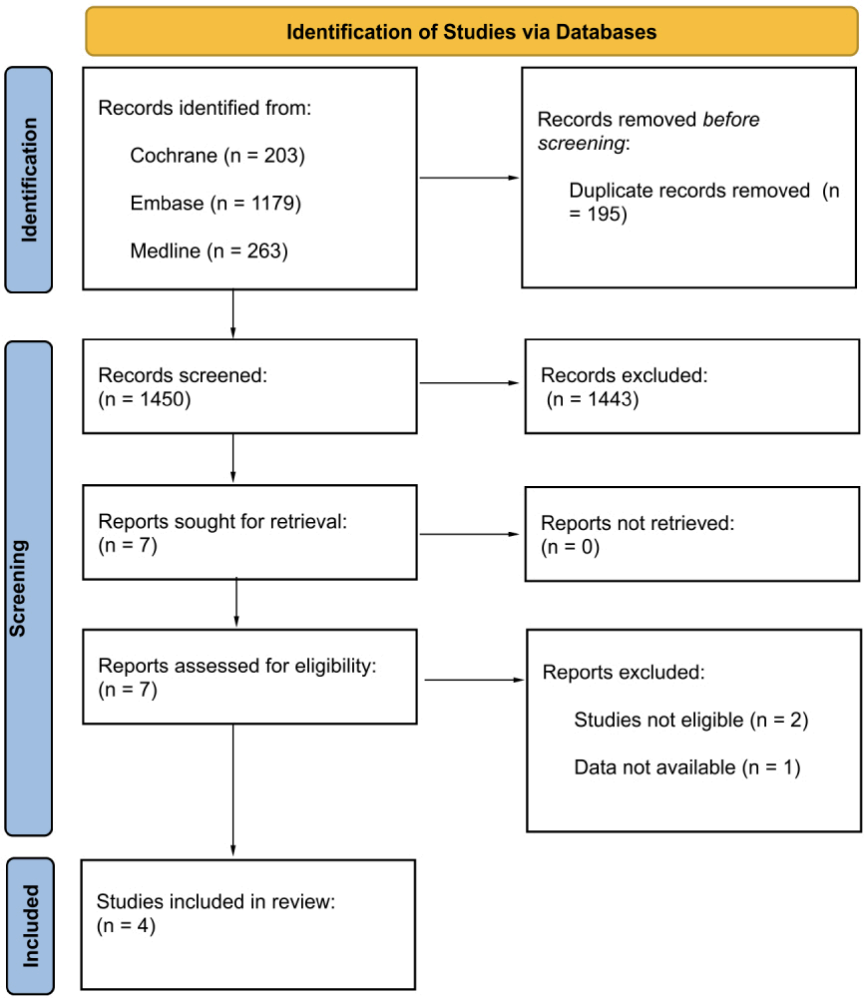
Study flow diagram in accordance with Preferred Reporting Items for Systematic Reviews and Meta-Analyses (PRISMA) guidelines *Note.* Adapted from “The PRISMA 2020 statement: an updated guideline for reporting systematic reviews” by Page MJ, McKenzie JE, Bossuyt PM, Boutron I, Hoffmann TC, Mulrow CD, et al. BMJ. 2021;372:n71 (https://doi.org/10.1136/bmj.n71). CC BY.

Exclusion criteria included studies not reporting these outcomes, trials with incomplete adverse event data, and non-randomized studies. Of the 1,645 records initially identified, 1,450 were screened after duplicates were removed. Four studies met the eligibility criteria: ICARIA-MM, IKEMA, IMROZ, and GMMG-HD7 (Figure 1 and Table 1).

**Table 1:** Characteristics of the studies included in the meta-analysis

**Study (author/year)**	**Study type**	**Number of patients (isatuximab group/control)**	**Cancer type**	**Drug (dose & duration)**	**Primary outcome measure**
ICARIA-MM [8] Attal et al. [13], 2019	Randomized, multicenter, open-label, phase 3 trial	154/153	Relapsed and refractory MM (RRMM)	Isatuximab (10 mg/kg intravenously) + pomalidomide (4 mg orally) + dexamethasone (40 mg orally or intravenously), in 28-day cycles.	Progression-free survival
IKEMA [9] Moreau et al. [14], 2021	Multicenter, open-label, randomized, phase 3 trial	179/123	RRMM	Isatuximab (10 mg/kg intravenously weekly for the first 4 weeks, then every 2 weeks) + carfilzomib (20 mg/m^2^ initially, followed by 56 mg/m^2^) + dexamethasone (20 mg orally or intravenously).	Progression-free survival
IMROZ [10] Facon et al. [15], 2024	Phase 3, open-label, multicenter, randomized trial	265/181	Newly diagnosed MM, ineligible for transplantation	Isatuximab (10 mg/kg intravenously) + bortezomib (1.3 mg/m^2^ subcutaneously) + lenalidomide (25 mg orally) + dexamethasone (20 mg orally or intravenously) in 6-week cycles.	Progression-free survival
GMMG-HD7 [11] Goldschmidt et al. [16], 2022	Phase 3, open-label, multicenter, randomized, active-controlled trial	331/329	Newly diagnosed, transplantation-eligible MM	Isatuximab (10 mg/kg intravenously) + lenalidomide (25 mg orally) + bortezomib (1.3 mg/m^2^ subcutaneously) + dexamethasone (20 mg orally) for 42-day cycles.	Minimal residual disease negativity

MM: multiple myeloma

Data were independently extracted by two reviewers (DTJ and HA), and discrepancies were resolved by another two reviewers (KZT and TWH). Extracted data included trial characteristics (study design, patient populations, and treatment regimens), and outcomes (incidence of pneumonia, URTIs, and VTE, categorized by any-grade and high-grade). The risk of bias for included studies was assessed using the Cochrane Risk of Bias Tool.

**Figure 2:**
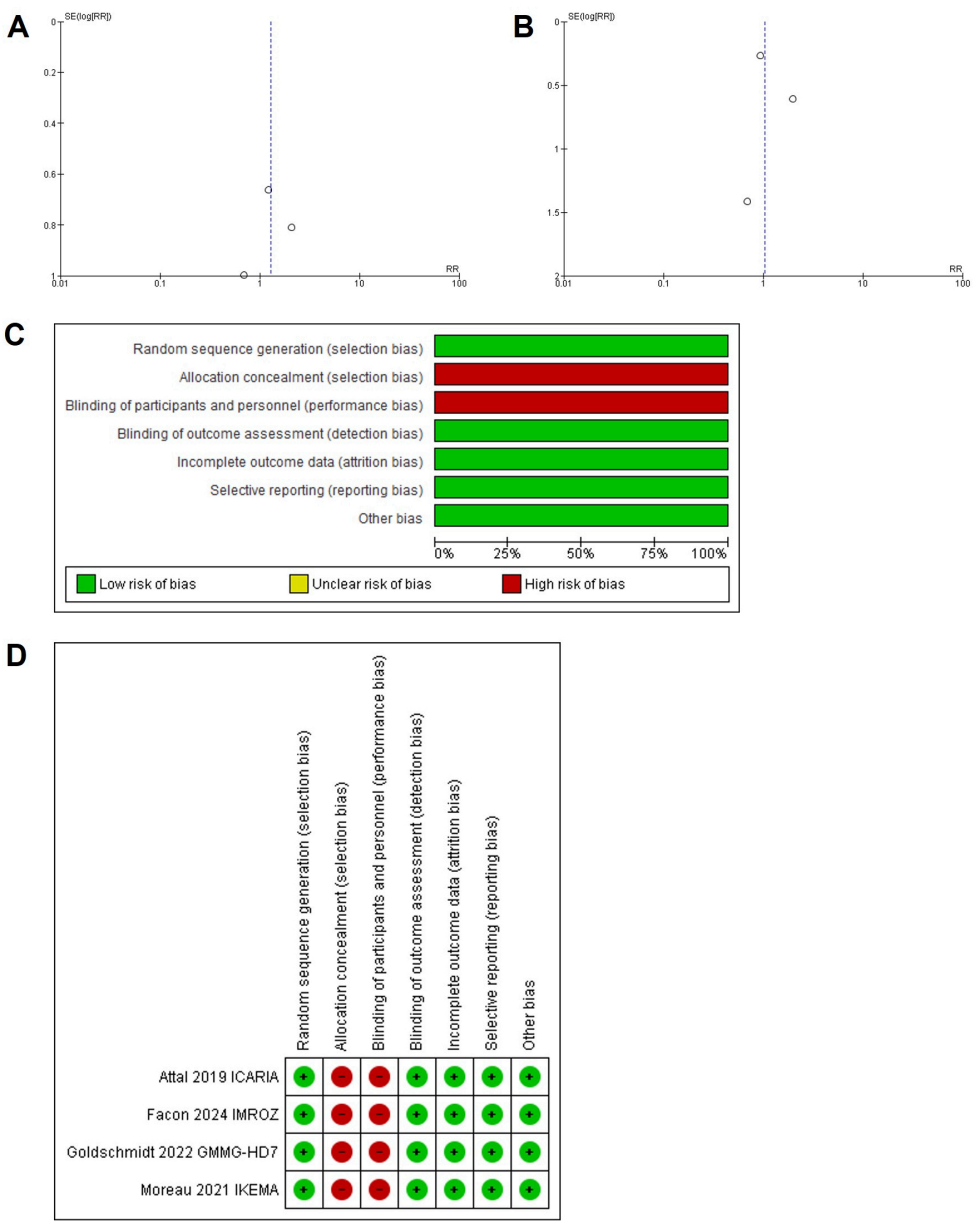
**Funnel plot of comparison: A. pneumonia and URTI, outcome; B. venous thromboembolism, outcome; and (C, D) risk of bias summary and graph**. URTIs: upper respiratory tract infections

The primary outcome was the incidence of pneumonia, URTIs, and VTE in the isatuximab and control groups. Pooled risk ratio (RR) and risk differences (RD) with 95% confidence intervals (CIs) were calculated for each outcome using the Mantel-Haenszel (MH) method. Heterogeneity was assessed using Cochran’s Q-statistic and the *I*^2^ statistic, with an *I*^2^ value > 50% considered indicative of substantial heterogeneity. Therefore, a random effects model was applied to account for clinical and methodological variability across trials. Sources of heterogeneity included differences in study design (e.g., open-label vs. double-blind), variations in patient populations (e.g., newly diagnosed vs. RRMM, age, comorbidities), and treatment protocols (e.g., variations in prophylactic antibiotic and thromboprophylaxis strategies, drug combinations, and dosing regimens). Statistical significance was defined as a *P*-value < 0.05. Funnel plots were generated to evaluate potential publication bias, and sensitivity analyses were conducted to test the robustness of the findings (Figure 2A, 2B, 2C, and 2D). All statistical analyses were performed using Cochrane RevMan software (version 5.3).

## Results

The characteristic features of the included studies are summarized in Table 1. A total of 1,044 patients from three phase III RCTs were eligible for analysis of pneumonia and URTIs: ICARIA-MM, IKEMA, and IMROZ. For the analysis of VTE, 1,403 patients from three RCTs were included: IKEMA, IMROZ, and GMMG-HD7. The ICARIA-MM trial compared isatuximab combined with pomalidomide and dexamethasone vs. pomalidomide and dexamethasone alone in patients with RRMM. The IKEMA trial evaluated isatuximab in combination with carfilzomib and dexamethasone compared to carfilzomib and dexamethasone alone in RRMM. The IMROZ and GMMG-HD7 trials focused on NDMM, with isatuximab combined with bortezomib-based regimens and lenalidomide plus dexamethasone, respectively.

Of the total population, 923 patients received isatuximab across the studies. The random effects model was applied to account for heterogeneity, as the *I*^2^ statistic indicated moderate variability among the included RCTs. Any-grade pneumonia was reported in 30.1% of patients in the isatuximab group compared with 23.2% in the control group, yielding an RR of 1.31 (95% CI: 1.06–1.61; *P* = 0.01). High-grade pneumonia occurred in 20.8% of the isatuximab group and 15.3% of the control group, with an RR of 1.38 (95% CI: 1.06–1.81; *P* = 0.02). The incidence of any-grade URTIs was 35.5% in the isatuximab group and 27.7% in the control group, but the pooled RR was not statistically significant (RR, 1.31; 95% CI: 0.96–1.78; *P* = 0.09). High-grade URTIs were rare, occurring in 2.2% of patients in the isatuximab group vs. 1.8% in the control group, with also a statistically not significant RR of 1.28 (95% CI: 0.53–3.13; *P* = 0.58). The incidence of VTE was 4.67% in the isatuximab group compared with 3.96% in the control group, with a pooled RR of 1.04 (95% CI: 0.65–1.68; *P* = 0.87).

**Figure 3:**
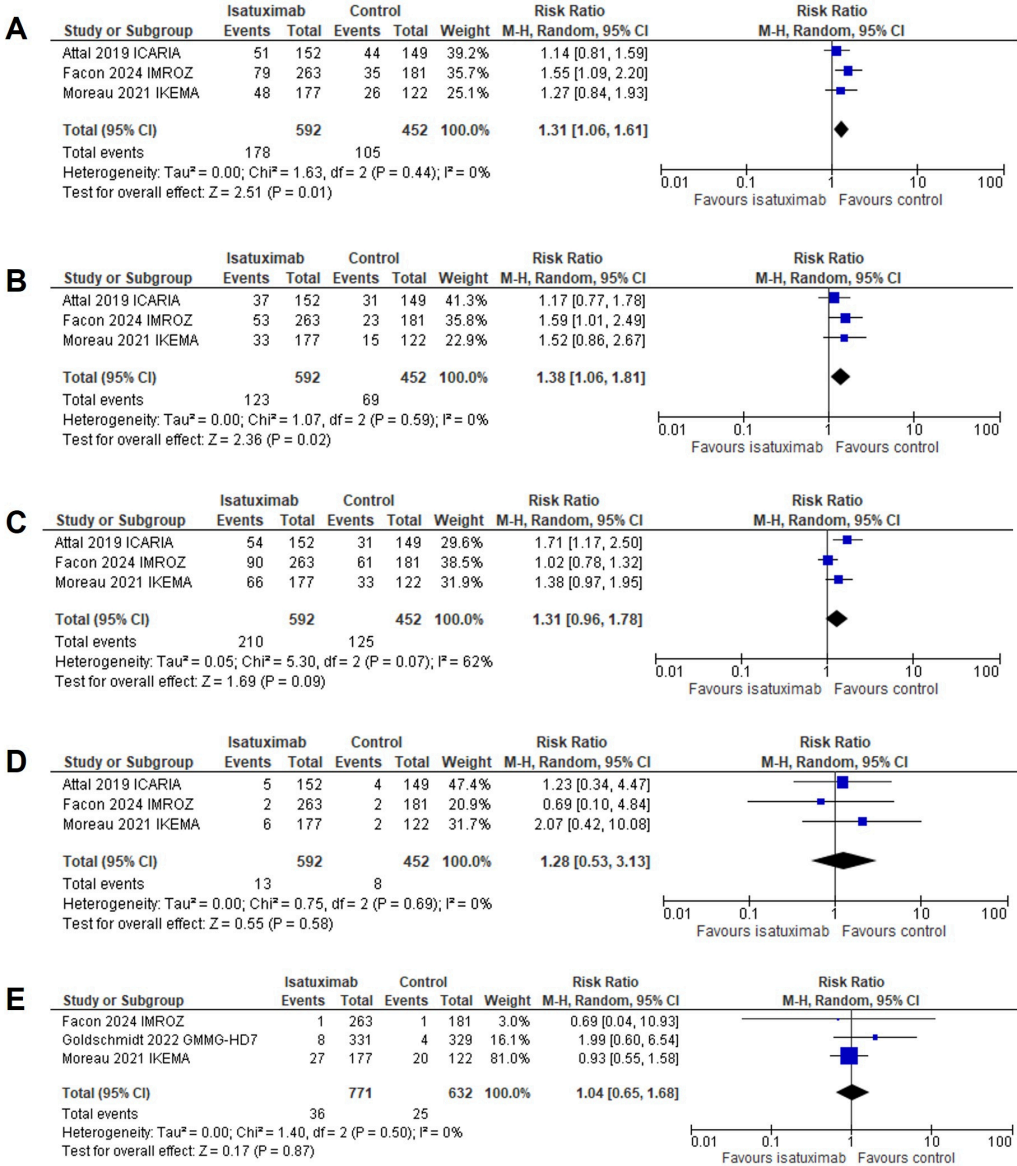
**Forest plots for (A) any-grade pneumonia, (B) high-grade pneumonia, (C) any-grade URTIs, (D) high-grade URTIs, and (E) VTE events**. URTIs: upper respiratory tract infections; VTE: venous thromboembolism; CI: confidence interval

Figure 3 provides forest plots summarizing the incidence of these adverse events. Any-grade pneumonia events occurred in 178 patients in the isatuximab arm vs. 105 in the control arm, with a RR of 1.31 (*P* = 0.01), indicating a statistically significant increase (Figure 3A). High-grade pneumonia events were reported in 123 patients in the isatuximab arm compared to 69 in the control arm, with a RR of 1.38 (*P* = 0.02), also showing a significant increase (Figure 3B). Any-grade URTIs were observed in 210 patients in the isatuximab arm vs. 125 in the control arm, with a RR of 1.31 (*P* = 0.09), suggesting a trend but not reaching statistical significance (Figure 3C). High-grade URTIs were reported in 13 patients in the isatuximab arm compared to 8 in the control arm, with a RR of 1.28 (*P* = 0.58), showing no significant difference (Figure 3D). VTE events were noted in 36 patients in the isatuximab arm compared to 25 in the control arm, with a RR of 1.04 (*P* = 0.87), indicating no significant difference in VTE risk (Figure 3E). Forest plots with results are shown in (Figure 3A–E).

## Discussion

Isatuximab is a CD38-targeting monoclonal antibody that exerts its anti-myeloma effects through multiple mechanisms, including antibody-dependent cellular cytotoxicity, complement-dependent cytotoxicity, antibody-dependent cellular phagocytosis, and direct induction of apoptosis. By binding to CD38, which is highly expressed on myeloma cells, isatuximab enhances immune-mediated destruction of malignant plasma cells and disrupts their survival pathways, demonstrating significant efficacy in RRMM, particularly when combined with immunomodulatory drugs, proteasome inhibitors, or corticosteroids [1, 2].

However, CD38 is also expressed on normal immune cells, including regulatory T cells (Tregs), natural killer (NK) cells, and B cells, which are essential for immune regulation and defense. CD38 plays a critical role in B-cell differentiation, neutrophil and monocyte chemotaxis, and T-cell activation. Its targeting by isatuximab results in B-cell depletion, reduced phagocytic activity, and impaired T-cell responses, all of which favor the development of infections. Furthermore, CD38 is involved in NAD^+^ metabolism and calcium signaling, critical for immune cell energy balance, and its disruption may exacerbate immune dysfunction. These immunosuppressive effects highlight the importance of infection surveillance, prophylactic strategies, and optimized supportive care in patients treated with isatuximab to reduce infection-related risks [11].

This meta-analysis revealed that adding isatuximab to standard MM regimens significantly increases the risk of both any-grade and high-grade pneumonia. The incidence of any-grade pneumonia was 30.1% in the isatuximab group compared to 23.2% in the control group, while high-grade pneumonia occurred in 20.8% of patients in the isatuximab group vs. 15.3% in the control group. These findings align with prior studies suggesting that isatuximab’s immunosuppressive effects may predispose patients to infections [3]. The pooled RR of 1.31 for any-grade pneumonia and 1.38 for high-grade pneumonia underscores the importance of vigilant infection monitoring and prophylactic strategies in patients receiving isatuximab-based therapies [4].

While the incidence of any-grade URTIs was higher in the isatuximab group compared to control groups, this difference did not reach statistical significance. The trend toward increased risk, however, warrants further investigation in larger, prospective studies to determine whether there is a true association. Notably, high-grade URTIs were rare and did not significantly differ between groups, indicating that while isatuximab may slightly increase the frequency of URTIs, these infections do not generally progress to severe forms requiring intensive care.

Antibiotic prophylaxis is a critical consideration for mitigating the infection risks associated with isatuximab-based regimens in MM patients. However, the uniform implementation of prophylactic antibiotics in MM clinical trials has been historically inconsistent, as reporting on their use has often been sparse or incomplete. Evidence from prior randomized trials demonstrates the efficacy of prophylactic strategies, such as levofloxacin administered during the first 12 weeks of therapy for NDMM, in significantly reducing febrile episodes and mortality without increasing healthcare-associated infections. Given that infection risks remain substantial across both frontline and RRMM settings, antibiotic prophylaxis should be considered for all treatment phases. Future studies are urgently needed to evaluate the long-term benefits of prophylaxis and its potential effects on antimicrobial resistance patterns. To improve clinical applicability, ongoing and future trials must provide consistent and transparent reporting on prophylactic antibiotic use [12].

VTE is a significant concern in MM due to disease-related hypercoagulability and treatment-induced factors, with reported incidences ranging from 3% to 12%. Certain regimens, such as lenalidomide combined with high-dose steroids, can increase VTE risk to as high as 26%. Our analysis found no significant increase in VTE incidence with isatuximab-based regimens compared to standard therapies, suggesting that isatuximab does not exacerbate thromboembolic risk despite the inherently high susceptibility in this patient population. Given this substantial baseline risk, primary pharmacological thromboprophylaxis with aspirin, LMWH, or direct oral anticoagulants (DOACs) is strongly recommended for all myeloma patients receiving antimyeloma treatment to mitigate thrombotic complications [9].

Several limitations of this meta-analysis should be noted. First, the heterogeneity among the included studies, particularly differences in treatment regimens and patient populations in different geographic regions, poses a challenge in generalizing the results. While the random effects model was employed to address variability, factors such as prior treatments, disease stage, and supportive care measures may have influenced the observed outcomes. Second, the limited number of phase III RCTs available for inclusion restricts the robustness of the findings, particularly for outcomes like high-grade URTIs and VTE. The relatively small sample size further reduces statistical power, potentially impacting the ability to detect significant differences for less frequent adverse events. Additionally, the pooled data may mask patient-level variables, such as age, performance status, or comorbidities, which could influence the risk of adverse events. Future studies with larger sample sizes, including multi-center trials and real-world data, are essential to validate these findings, investigate these variations, and improve the generalizability of treatment outcomes.

Despite the increased risk of pneumonia, isatuximab-based regimens have demonstrated significant efficacy in improving minimal residual disease negativity, very good partial response or better, and complete response rates, particularly in NDMM patients [13–16]. These findings underscore the potential of isatuximab to deliver superior disease control when integrated into first-line and RRMM treatment protocols.

In conclusion, our meta-analysis highlighted the safety profile of isatuximab when combined with standard therapies for MM. While its use significantly increased the risk of any-grade and high-grade pneumonia, the risks of URTIs and VTE were not significantly different from control regimens. These findings emphasize the necessity of routine infection control measures, including antibiotic prophylaxis, vaccination, and close monitoring to mitigate pneumonia risks. Additionally, primary pharmacological thromboprophylaxis with aspirin, LMWH, or DOACs should remain a cornerstone of care to address the elevated thrombotic risk in this patient population. Despite these safety considerations, isatuximab-based regimens continue to demonstrate significant efficacy, improving outcomes such as minimal residual disease negativity and response rates in both newly diagnosed and relapsed/refractory patients.

## Abbreviations

CI: confidence interval
DOACs: direct oral anticoagulants
LMWH: low molecular weight heparin
MH: Mantel-Haenszel
MM: multiple myeloma
NDMM: newly diagnosed multiple myeloma
PRISMA: Preferred Reporting Items for Systematic Reviews and Meta-Analyses
RCTs: randomized controlled trials
RD: risk differences
RR: risk ratio
RRMM: relapsed and refractory multiple myeloma
URTIs: upper respiratory tract infections
VTE: venous thromboembolism

## Declarations

### Availability of data and materials

All datasets analyzed for this study are included in the manuscript.

### Copyright

© The Author(s) 2025.
